# Phenological Plant Pattern in the Topographic Complex Karstic Landscape of the Northern Dinaric Alps

**DOI:** 10.3390/plants14071093

**Published:** 2025-04-01

**Authors:** Aljaž Jakob, Mateja Breg Valjavec, Andraž Čarni

**Affiliations:** 1 Jovan Hadži Institute of Biology, Research Centre of the Slovenian Academy of Sciences and Arts, Novi trg 2, 1000 Ljubljana, Slovenia; aljaz.jakob@zrc-sazu.si; 2 Department of Biology, Biotechnical Faculty, University of Ljubljana, Večna pot 111, 1000 Ljubljana, Slovenia; 3 Anton Melik Geographical Institute, Research Centre of the Slovenian Academy of Sciences and Arts, Novi trg 2, 1000 Ljubljana, Slovenia; mateja.breg@zrc-sazu.si; 4 School for Viticulture and Enology, University of Nova Gorica, Vipavska 13, 5000 Nova Gorica, Slovenia

**Keywords:** climate change, doline, ecology, flowering, forest, karst, K-means, phenology, topography, vegetation

## Abstract

Vegetation phenology has lately gained attention in the context of studying human-induced climate change and its effects on terrestrial ecosystems. It is typically studied on various regional and temporal scales. This research focused on the microscale in dolines on the Northernmost part of the Dinaric Alps. The aim was to determine the timing of flowering onset and relate it to topographic and ecological conditions. We studied (1) the floristic gradient along N–W transects divided in 2 m × 2 m plots, from top slopes to the bottom of dolines, and identified discrete groups in relation to this gradient and (2) provided their diagnostic species and communities. The results indicate that the early spring onset of flowering of ground vegetation in the bottom and lower slopes of dolines is stimulated by high spring moisture and nutrient availability, as well as the open canopy of the mesophilous deciduous forests. The flowering onset on the upper slopes and karst plateau starts later, which is due to the precipitation peak in May/June and higher temperatures and light availability of the open canopy of thermophilous deciduous forests. The delayed onset of flowering in late summer in rocky crevices and rocky places is due to a particular physiology stimulated by the harsh site conditions. The phenology pattern along the doline topographic gradient is inverse to general patterns in vegetation phenology. Further study on the role of doline soils should be made to study their impact on phenology.

## 1. Introduction

Phenology is the study of natural phenomena that recur periodically in living organisms and their relationship to climatic seasonal changes [1]. In recent decades, the study of vegetation phenology has gained attention, especially in the context of studying human-induced climate change and its effects on terrestrial ecosystems [2,3]. Temperature is often considered the main factor for phenological changes, but several studies have shown that other factors, such as precipitation, also have an important influence [4]. Temperature is the dominant factor for spring phenology in cold regions, while precipitation, radiation, and temperature co-determine spring phenology in warm regions [5].

Phenology is typically studied on various regional and temporal scales [6]. However, our research focused on the microscale since it has been shown that the distribution patterns of functional traits (i.e., the phenological spectrum) reflect microclimatic variations in a topographically complex karst environment [7,8]. At microsites, plants are in physical contact, and their distribution patterns and occurrence result from their interactions and the environmental characteristics of the site in which they occur [9]. This pattern can differ in areas under different macroclimates [10].

Our research took place in dolines on the Karst Plateau, where the topographic complexity favors the maintenance of a great diversity of microhabitats, and different types of vegetation can occur [11]. Dolines are considered microrefugia, places with special environmental conditions that facilitate the survival of species during environmental changes and exhibit unique eco-evolutionary dynamics [12]. These areas, which can harbor a high density of vegetation types, are more likely to maintain the current flora and vegetation in the warmer future [13] since their ability to create steep ecological gradients is at least partly associated with inertia in environmental parameters [14].

In recent years, intensive investigations have been carried out into areas that could provide safe havens for biota under climate change [15]. As dolines are part of these areas, they have attracted the attention of researchers, and many studies have been carried out [16,17,18]. Recently, it has been found that early flowering of plants can be observed on the north-facing slopes of dolines in Northern Hungary, and these plants can, thus, provide food also for other biotas [12]. It is important to focus research on this topic and find reasons for this pattern, which contradicts common knowledge.

The aim was to determine the timing of the flowering onset along the gradient and relate it to topographical (bare rock, sky-view factor, and depth of the plot) and ecological conditions (light, temperature, nutrients, moisture, and soil reaction). Accordingly, we investigated the vegetation on a gradient from the doline rim (coinciding with the plateau) to the bottom of dolines, determined discrete groups along this gradient (1), calculated the diagnostic species of these groups, and determined the position of forest communities along the gradient (2).

## 2. Results

### 2.1. Presentation of Floristic Gradient and Discrete Groups

**Figure 1:**
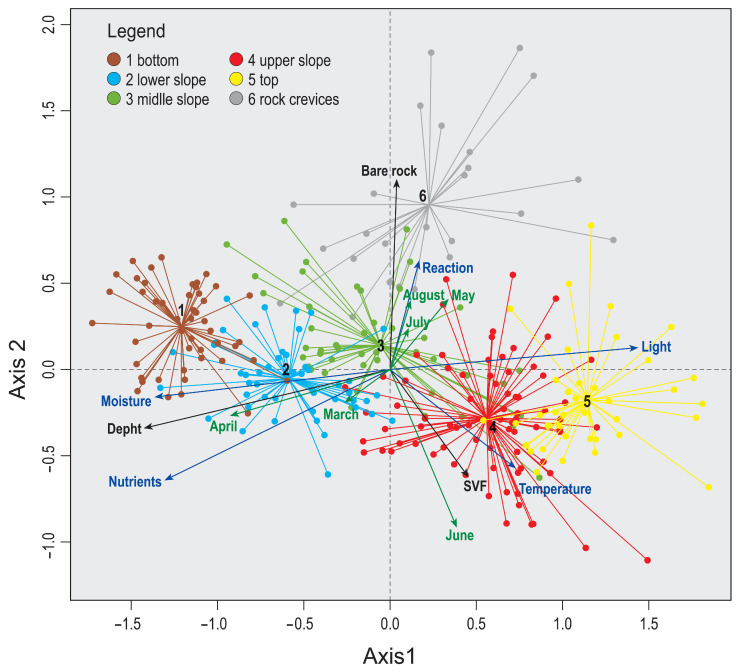
DCA presenting the six groups identified by K-means, eigenvalues 0.4481 and 0.1988. The topographical variables (depth, SVF, bare rock), EIV (light, temperature, moisture, nutrients, soil reaction), and different months (March–August) are passively projected on the ordination plane. Legend: 1—bottom; 2—lower slope; 3—middle; 4—upper slope; 5—karstic plateau; 6—vegetation of rock crevices.

**Figure 2:**
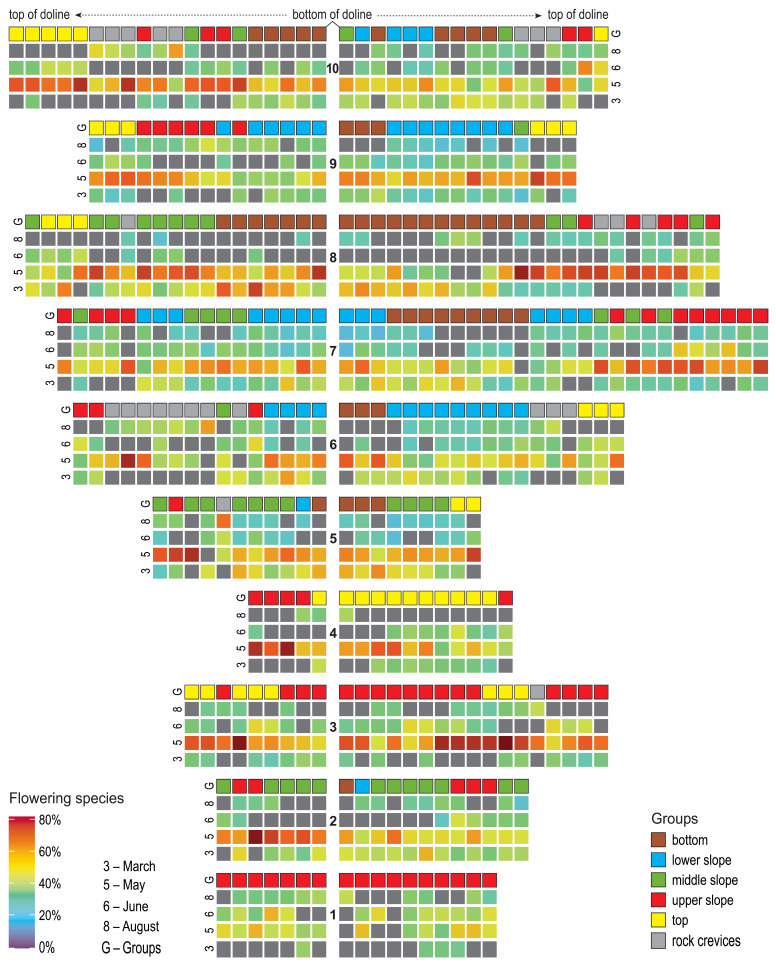
Plots in transects colored according to the result of analysis. Transects in the figure are orientated from south (**left**) to north (**right**); there is an indication of transect at the lowest point of the transect (**bottom**).

**Figure 3:**
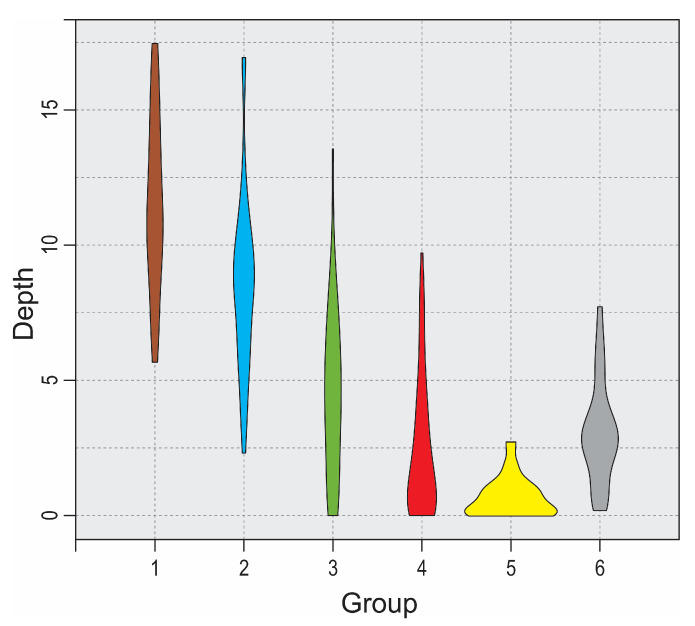
Violin plots of depth of sample plots (from the top edge of the dolines). Legend: 1—bottom; 2—lower slope; 3—middle; 4—upper slope; 5—karstic plateau; 6—vegetation of rock crevices.

The DCA analysis revealed the longest floristic gradients in the data matrix, presented by axes 1 and 2 (Figure 1). Silhouette analysis suggested that the optimal division of the matrix would be into six groups, and there was no need to shift plots (Figure S1). Six groups were plotted on the DCA diagram with a passive projection of topographical variables, EIV, and flowering onset (Figure 1). The first axis correlated strongly with the depth of the individual plot and nutrients, moisture, and light, while the second axis correlated with bare rock and temperature (Figure 1, Table 1). Flowering onsets in March, June, May, and August had the highest explanatory power on floristic composition (Table 2), and these are presented in Figure 2. Since the depth of the plots is the most important distinguishing feature, the six groups are presented using violin plots (Figure 3).

### 2.2. Results of Diagnostic Species of Groups Calculations and Assignment of Plots to Communities

The synoptic table (Table 3) shows the diagnostic species of groups. *Asarum europaeum, Galanthus nivalis,* and *Anemone nemorosa* appear at the bottom of the deepest dolines (group 1); *Symphytum tuberosum, Fragaria moschata,* and *Primula vulgaris* appear on the lower slope (group 2); in the middle group (group 3), *Acer monspessulanum, A. campestre,* and *Melittis melissophyllum* are found; the upper slope (group 4) is characterized by *Vincetoxium hirundinaria, Lathyrus sylvestris,* and *Genista tincoria*; at the top, appear *Cotinus coggygria, Brachypodium rupestre,* and *Carex humilis* (group 5), and finally, group 6 represents chasmophytic vegetation of small cliffs and rocks in the dolines, consisting of *Moehringia muscosa, Asplenium ruta-muraria,* and *Campanula pyramidalis* (Table 3 and Table S1).

The groups correspond to the following forests (only forests appearing in more than three plots within each group are mentioned): bottom (group 1): oak–hornbeam forests (*Asaro europaei–Carpinetum betuli*), mesophilous ravine forests (*Corydalido ochroleucae-Aceretum pseudoplatani*), sessile oak forests (*Seslerio autumnalis–Quercetum petraeae*), thermophilous ravine forests (*Paeonio officinalis–Tilietum platyphylli*); lower slope (group 2): sessile oak forests (*Seslerio autumnalis–Quercetum petraeae*), oak–hornbeam forests (*Asaro europaei–Carpinetum betuli*), thermophilous ravine forests (*Paeonio officinalis–Tilietum platyphylli*), Turkey oak forests (*Seslerio autumnalis–Quercetum cerridis*); middle slope (group 3): Turkey oak forests (*Seslerio autumnalis–Quercetum cerridis*), mesophilous ravine forests (*Corydalido ochroleucae–Aceretum pseudoplatani*), hophornbeam–pubescent oak forests (*Aristolochio luteae–Quercetum pubescentis*); upper slope (group 4): Turkey oak forests (*Seslerio autumnalis–Quercetum cerridis*), hophornbeam–pubescent oak forests (*Aristolochio luteae–Quercetum pubescentis)*, shrub communities (*Frangulo rupestris-Prunetum mahalebis*); karstic plateau/top (group 5): hophornbeam–pubescent oak forests (*Aristolochio luteae–Quercetum pubescentis*), shrub communities (*Frangulo rupestris-Prunetum mahalebis*); rock crevices (group 6) vegetation of rock crevices (*Asplenietum trichomanis–rutae–murariae*) (Table 3 and Table S2).

### 2.3. Relation of Time of Flowering Onset to Topographical and Ecological Variables

The flowering onset in March correlates with the depth of the plots in dolines, an increased amount of nutrients, and higher moisture. The onset in May, when most species flower, shows that these plots appear on the upper part of the slope, with higher values of SVF. Flowering in June is closely related to the upper slope and top, high SVF value, low percentage of bare rock on the surface, and high light intensity. In August, the onset of flowering of plants in plots with a high proportion of bare rock on the surface and lower SVF values can be observed. Flowering onset in April and July is not commented on since they are of transitional character (Table 4).

In Figure 2, the presentation of the occurrence of different groups (line G in the figure) shows that in shallow dolines (up to 8 m), group features for the bottom and the lower slope are not found. In these dolines, an early flowering onset (in March) cannot be observed. In the shallow dolines, however, the flowering onset in May appears at the bottom, while in the deeper dolines, the flowering onset in May appears on the slopes. A beginning of flowering in June can also be observed on the slopes and the karst plateau. Flowering in August takes place in plots on rock crevices and rocky surfaces.

## 3. Discussion

### 3.1. Floristic Gradient and Discrete Groups

We identified six groups of sample plots along transects in dolines using the K-means method, which minimizes the distances within the groups. The decision was made according to the results of silhouette analysis, which visualized the distance within a cluster and gave information about its homogeneity [19,20]. The diagram of DCA showed that these six groups were well arranged along the floristic gradients that correlate with topographical variables and ecological site conditions. This division reflects the depth of plots (Figure 3) and is also ecologically sound, as can be seen from the diagnostic species (Table 3) and the appearance of the forest communities along the transects (Table S2).

When this classification, which produces spherical or regularly shaped clusters, is compared with the commonly used classification based on similarity, which produces clusters of irregular shape in multivariate species space (e.g., TWINSPAN c.f. [21]), it can be concluded that K-means generates groups with sharp boundaries. Hierarchical clustering always divides groups and does not detect transitional groups. In this way, a transitional group between the lower and upper slope (i.e., middle group) was discovered, which combined elements of mesophilic forests occurring in the bottom and lower slopes and thermophilic forests occurring on the upper slopes and karst plateau [11,22].

### 3.2. Diagnostic Species of Groups and Assignment to Forest Communities

We established six groups that corresponded to their location in the doline. At the bottom, appear mesic forests dominated by hornbeam (*Carpinus betulus*), sycamore maple (*Acer pseudoplatanus*), and sessile oak (*Quercus petraea*); on the lower slopes, can be found forests dominated by lime *(Tilia cordata*); on upper slopes, appear forests dominated by Turkey oak (*Quercus cerris*) and scrub (dominated by *Frangula rupestris, Prunus mahaleb*), and on the karst plateau thrive forests dominated by hophornbeam (*Ostrya carpinifolia*) and pubescent oak (*Quercus pubescens*). These forests can be assigned to the following habitats: *Carpinus betulus* dominated forests to T1E–*Capinus betulus* and *Quercus* mesic deciduous forests; *Acer psedoplatanus* and *Tilia* dominated forests to T1F–ravine forests, and the remaining forests to T1E–temperate and sub-Mediterranean thermophilic deciduous forests [23]. Mesophilic deciduous forests can be distinguished, dominated by hornbeam, sessile oak, and lime assigned to the *Carpino-Fagetea* class, and thermophilic deciduous forests classified within *Quercetea pubescentis* [24]. In the context of climatic changes, it is expected that sclerophyllous forests dominated by holm oak (*Quercus ilex*) will develop on the Karst Plateau, while the current vegetation will find shelter in the depressions (dolines, collapse dolines) [25,26].

It is difficult to predict the potential sites of forests that appear in dolines in the wider region. According to Poldini [27], the temperature drops by 7 degrees in 100 m, depending on the depth of the doline. Considering an altitudinal drop of 0.6 degrees in 100 m and a depth of doline of 20 m, such vegetation could be expected somewhere around 250 m above the research area. Hornbeam forests also appear at that altitude [28], but such conclusions, without an in-depth study of site conditions and vegetation, are premature.

### 3.3. Time of Flowering Onset in Relation to Topographical and Ecological Variables

The data on flowering onset were obtained from a database that is relevant to a broader region [29,30] and appended by the local one [31]. The results would be more reliable if data were recorded on the research site. It must also be taken into consideration that flowering onset is also initiated by environmental factors [32]. The flowering onset, even within positions in a single doline, thus varies by a few days, and interannual variation in phenological stages also exists, as well as changes due to climatic changes [33]. All this causes some limitations to our study, but we think that the data are robust enough, and these limitations do not significantly influence the results.

The analysis showed that species begin to flower in early spring at the bottom and on the lower slopes of the dolines. These habitats are moist and nutrient-rich. The relatively cool temperatures during winter and high humidity due to water accumulation at the bottom, as well as snowmelt at the beginning of spring, enable rapid mineralization; at the same time, dolines are also a trap for soils and a deep layer of relatively moist soil can be found in the bottom [34,35]. It must be borne in mind that hornbeam (*Carpinus betulus*) forms a dense canopy and is considered a superior competitor on mesic and fertile soils so that shade-tolerant and nutrient-loving plants can thrive in the herb layer. The high humidity in ravine forests is accompanied by a relatively rapid decomposition of their nutrient-rich litter. Due to the closed canopy of these forests, the species in the ground layer are forced to flower quite early [27,36,37].

The distribution of plant communities in the doline is not random, and two of the most significant factors influencing the distribution are depth and SVF. Both factors significantly alter the environment: deeper plots generally have deeper soils [34], and SVF is a measure of total light availability [38]. These two factors are not completely independent, as deeper plots also have smaller SVF due to the slopes of the doline obscuring the true horizon [39]. However, SVF is further very dependent on doline shape and steepness, not only the depth. Deeper soils at the bottom of dolines cause trees to grow higher, further restricting light availability there. Low light availability reduces the amount of energy the ground receives, causing it to be cooler and deeper soils retain moisture, enabling more species from typically moister, more continental regions to thrive at the bottom of dolines [40,41], which are, otherwise, absent from the region (i.e., *Cardamine enneaphyllos*, *Lathraea squamaria*). These species are adapted to exploit the brief period in early spring when light availability in mesic forests is adequate before the trees leaf out.

On the slopes of dolines, there are Turkey and pubescent oak forests, whose open canopy allows for the growth of a rich herbal layer. The SVF is greater here, and the soils are shallower, in parts composed of rolling stones and bare rocks, as well as pockets of deeper soil [11,21,41]. The doline slopes receive more light. The higher light availability reduces the advantage of early flowering spring ephemerals, which are outcompeted by larger herbs and grasses (i.e., *Paeonia officinalis, Sesleria autumnalis*). The general peak of flowering in this part of Karst is in late spring before the summer heat and drought. In most of our plots, this is reflected by the highest proportion of plant species beginning the flowering in May; however, this peak is most obvious on middle and upper slopes, where the proportion can reach 80%.

In May, plants begin to flower throughout the doline. The vegetation benefits from the high temperatures and high rainfall at this time. Since rainfall peaks around May/June in most years, there is no water stress for the plants, although the bedrock is highly permeable and the soils are generally thin, so only a small proportion of water is stored in the soil reserves. Between 30 and 40% of the plant species start to flower at this time. The plots with the highest proportion of such species are mostly thermophilous deciduous forests, which have an open canopy. The plant species have more conservative resource utilization strategies (long-lived leaves and a low photosynthetic rate), but the plants are taller and have larger seeds [27,36].

On the top and around the dolines, there is the most light available (SVF is the highest), and the soils are shallower; in places, barren bedrock is exposed on the surface. The trees are the shortest here, and the canopy can be interrupted, allowing the patches of grassland to develop. The shallow soils cannot retain the water, which is drained underground through pathways in the limestone bedrock, and high insolation causes the top soils to dry out frequently during the growing season. These parts of dolines have the most Mediterranean character. Dry and bright conditions enable rich communities of many graminoids to develop. Many of these species are wind-pollinated and start flowering at the start of the dry period in June [42].

In June, the highest proportion of plants begins to flower in the upper part and on the Karst Plateau, where a high SVF can be found. Evidence exists that high light availability delays the period of flowering. Many caespitose species (*Brachypodium rupestre, Festuca heterophylla*) that build closed stands are presented in these habitats [36,41,43]. This late onset of flowering may also be associated with a higher proportion of graminoid species, which are pollinated by wind. In wind-pollinated species, pollination success is higher in sunny and dry conditions. In June, the peak of rain has passed, and sun irradiation increases, making conditions for wind pollination significantly better [44].

The beginning of flowering in rock crevices can be seen in August. These plants are slow-growing, stress-tolerant rosulate hemicryptophytes. The sites are very rocky but have a limited sky-view factor. The stands are dry, and drought delays the flowering onset due to physiological processes [1,41,45]. Late summer and autumn flowerings are affordable for plants that can complete the cycle of flowering and seed maturation quickly, such as the annual *Geranium robertianum* or the biennial *Campanula pyramidalis*. Some of these chasmophytes complete the cycle before the summer drought (*Moehringia muscosa*), but certain plants are capable of completing it toward the end of the season, after the summer drought, and before the autumn rain (*Campanula pyramidalis*) [46,47].

The flowering onset in early spring at the bottom of deeper dolines is initiated by moisture and nutrients. Future climate scenarios with rising temperatures predict that drought will be the main threat to forests [48,49]. In the coming years, a “Mediterraneanization” of climate can be expected; it will become warmer and drier [50]. Plants will migrate to refugia with lower temperatures and higher moisture [7]. In our case, doline represents a microrefugium for mesic plants, but it is a question of how plants from thermophilic deciduous forests growing on shallow soils on tops and slopes would react to edaphic and light conditions deeper in the dolines [51], should the climate change enough to force significant vegetation shifts.

Dolines have been shown to be effective microrefugia for plant species [7] and communities [11]. They modify the environment in several ways: by providing high geodiversity in available microhabitats, modulating the availability of light, water, and nutrients, as well as edaphic conditions. It is tempting to consider how dolines, with their diverse habitats, may be able to mitigate the effect of climate change, as they have acted following global warming–there are several locally relict species associated with karst depressions in the area (i.e., *Primula auricula*) [52] and beyond (i.e., *Stachys alpina* in Hungary [18]). While doline can, indeed, mitigate the effect of increased temperatures and new precipitation regimes, it is also important to consider that these abilities are also connected with light availability and soil conditions, which will be limiting to many plants. From our research, we know that *Paeonia officinalis* cannot grow at the bottom of dolines, if SVF is not high enough. It remains to be seen whether this is due to total light availability (a factor also influenced by a tree-canopy structure) or the time between the appearance and disappearance of the sun in the visible sky (a factor dependent only on doline shape). If the climate in the area becomes too dry and hot, it may not be able to recede deeper into dolines.

## 4. Materials and Methods

### 4.1. Area of Research

**Figure 4:**
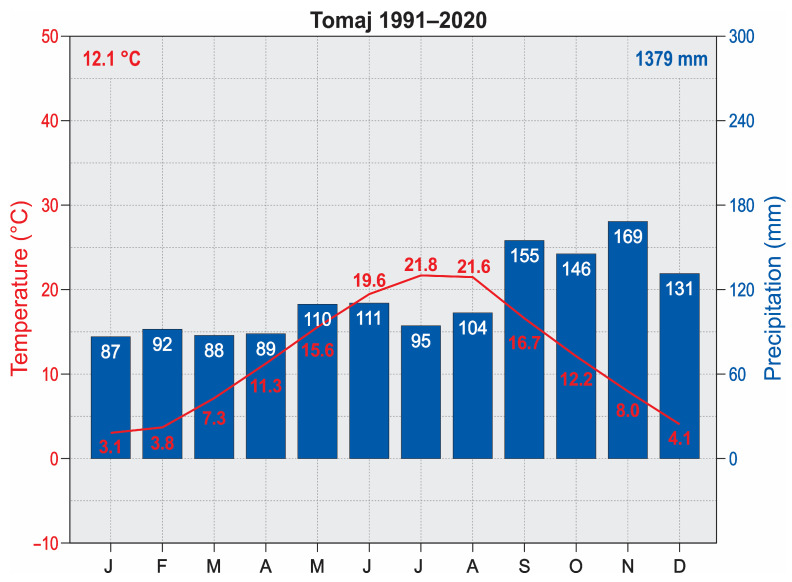
Climatic diagram of Tomaj (Source Slovenian Environment Agency).

This study took place on the Karst Plateau (350 km^2^), a limestone karst plateau located above the Bay of Trieste in the northernmost part of the Adriatic Sea at an altitude of 300–500 m. It is an uplifted and slightly leveled corrosion plain in the NW part of the Dinaric Alps [53]. The climate is transitional between Mediterranean and continental (i.e., sub-Mediterranean), with rainy, cool winters and hot summers. The annual rainfall is about 1400 mm, and the average annual temperature is about 12 °C (Figure 4). The Karst Plateau consists of karstified Mesozoic limestone, covered mainly by rendzinas and cambisols. The zonal forests in the area are dominated by hophornbeam (*Ostrya carpinifolia*), pubescent oak (*Quercus pubescens*), and manna ash (*Fraxinus ornus*) [54,55,56].

The object of research was dolines. They are natural, enclosed depressions formed by the dissolution of carbonate rock. They range in diameter from a few meters to several hundred meters and are slightly sloping to vertical on the sides. Their depth varies from a few meters to several tens of meters. Microrefugia occur in dolines because the ecological and climatic conditions change due to the concavity of the terrain [57,58,59].

In order to exclude the effects of disturbances (tree cutting, livestock grazing, agriculture), only dolines in forests were selected. We selected forests closely resembling potential natural vegetation, which is the result of climatic and edaphic conditions [60,61].

### 4.2. Fieldwork

The transect method was used to investigate the vegetation in the dolines. We sampled 10 dolines of various depths (Table 5). The elaborated dolines were selected in the same area to ensure the same macroclimatic conditions. We took continuous line transects with 2 m × 2 m plots from the northern edge of the doline over the bottom to the southern edge. The transects varied in length depending on the size of the dolines. All vascular plants in the ground layer were recorded and their cover was visually estimated according to the standard Central European 7-degree scale [62] (Table S1). The samples were recorded in the period May/June 2020. At the same time, we estimated the percentage of bare rock in each plot (Table S2). We used Lidar technology to establish the topographical variables [11,63].

### 4.3. Analysis

#### 4.3.1. Method of Analysis of Floristic Gradient and Establishment of Discrete Groups

K-means analysis was used to cluster sample plots. A square root transformation of the percentage coverage values and Hellinger transformation were performed. We set the program to 20 starts and 5 samples to define the starting centroid. The number of groups was evaluated using silhouette analysis (using the Bray–Curtis distance) [11,20,22].

The calculations were performed using the K-means function in the R package vegan [64] and silhouette-based reallocation methods using the function remos [20] in the “cluster” program [65], both running in R environment [66] integrated in the program JUICE [67].

The ability of Lidar to obtain high-resolution 3D data creates the potential to calculate sky-view factors (SVF) from the derived digital elevation model (DEM). The SVF is a measure of the amount of sky visible at a particular location: it is a dimensionless parameter, ranging from 0 (obscured view) to 1 (unobscured view). The depth of each sampled plot, i.e., the altitudinal distance from the highest plot in each individual transect (top) to the sample plot, was calculated. Calculations were made by the program Arc Info [68].

The depth of the plots in each group is presented in the form of violin plots, which combine aspects of a box plot and a kernel density plot. They show the distribution of variables across different communities, with the main features being the density of the data, the range, and the central tendency. They were created using the package ggplot2 in the R environment [66,69].

The following variables are grouped together in our text under the term topographical variables: depth of the plot, percentage of bare rock on the surface, and sky-view factor (SVF).

EIVs were used for the ecological interpretation of the vegetation pattern [70,71]. We calculated the mean EIV (light, moisture, nutrients, reaction, temperature) for each vegetation plot [72].

#### 4.3.2. Calculations of Diagnostic Species of Groups and Assignment of Plots to Communities

The diagnostic species of each group were defined by calculating the fidelity of each species to each group using the φ-coefficient as the fidelity measure [73], whereby species with a φ-value over 0.20 were considered diagnostic. The φ-coefficient was calculated for an equalized size of clusters. Species whose concentration of occurrence in the vegetation plots of a particular group was not significant at *p* < 0.05 (Fisher’s exact test) were excluded from the set of diagnostic species [74]. The other species were arranged by declining presence (Table 3 and Table S1). We assigned plots to the forest communities (Table S2) [11].

#### 4.3.3. Calculation of Time of Flowering Onset and Its Relation to Topographical and Ecological Variables

The information on the flowering onset (in months) for each plant species was provided from the BIOLFLOR database [29,30]. The start of flowering in February and March was combined into one category (March), as well as August and September (August). The missing values were provided from the local flora [31]. The percentage of plant species starting to flower in each month was calculated.

The topographical variables, EIV and month of flowering onset, were passively projected onto a DCA diagram representing the first two axes of the Detrended Correspondence Analysis [75].

The Spearman correlation coefficient was calculated between the first two DCA axes and topographical variables and EIV, as well as between the proportion of flowering onset in each month and topographical variables and EIV. Due to the circularity of the EIV, the function envfit.iv was used in the R environment to calculate the modified permutation test for the correlation with the EIV. The parametric significance was calculated assuming a normal distribution of the data, the permutation significance assuming that there is no relationship between EIV and species composition, and the modified permutation considering this relationship with a random distribution of EIV among species. The correlations with EIV were calculated and analyzed using the JUICE program [66,67,76,77].

Canonical correspondence analysis (CCA) was used with a Monte Carlo test with 999 unrestricted permutations to test the explanatory power of flowering onset in different months on the floristic composition [78]. The plots in the transects were colored according to the outcome of the K-means analysis and the proportion of species with the onset of flowering in March, May, June, and August to show the pattern of distribution along transects in the dolines.

## 5. Conclusions

The phenology pattern along a topographic gradient in the dolines is inverse to general patterns in vegetation phenology, where flowering onset is dependent on high temperature [79]. There are indications that precipitation, radiation, and temperature could co-determine spring phenology in warm regions [5]. However, our findings do not match these results, as it was found that the crucial factors for the early flowering onset in the bottom of dolines were humidity and nutrients. As this is one of the first studies on plant phenology in dolines, further studies are needed to establish the impact of soils accumulated at the bottom of dolines on phenology.

## Figures and Tables

**Table 1:** Correlation of the first two DCA axes with topographical variables and EIV. Legend: r—correlation coefficient; p.par—parametric correlation, p.perm.—with permutation test and p.modif—with modified permutation test; calculated probability in interval: ‘***’ 0.001 ‘**’ 0.01 ‘*’ 0.05; p.perm and p.modif. do not apply to topographical variables.

DCA1	r	p.par.	p.perm.	p.modif.	DCA2	r	p.par.	p.perm.	p.modif.
Depth of plots	−0.997	***			Depth of plots	0.318	***		
SVF	0.447	***			SVF	−0.408	***		
Bare rock	−0.025	***			Bare rock	0.659	***		
Moisture	−0.765	***	**	**	Moisture	0.208	***	**	-
Nutrients	−0.829	***	**	**	Nutrients	0.182	**	**	-
Light	0.85	***	**	**	Light	−0.331	***	**	-
Temperature	0.507	***	**	-	Temperature	−0.393	***	**	*
Reaction	0.109	-	-	-	Reaction	0.268	***	**	-

**Table 2:** Explanatory power of flowering onset on the floristic composition calculated by Canonical correspondence analysis (CCA). Legend: PTV—percentage of total variance; P—calculated probability; F—statistical significance.

Month	PTV	*p* Value	F Stat.
March	2.7	0.001	8
June	2.3	0.001	6.6
May	1.5	0.001	4.4
August	1.4	0.001	4
July	1.3	0.001	3.9
April	1.2	0.001	3.4

**Table 3:** Synoptic table of sample plots according to groups. Species are represented by percentage presence and fidelity multiplied by 100 (in superscript). Diagnostic species are framed. Only species that reach a fidelity of 20 and some of the most common species are presented in the table. Legend: 1—bottom; 2—lower slope; 3—middle slope; 4—upper slope; 5—karstic plateau; 6—vegetation of rock crevices.

Group No.	1		2		3		4		5		6	
Number of plots	50		48		48		76		39		25	
Asarum europaeum	44	^35.3^	19	^4^	21	^6.6^	1	^---^	.	^---^	8	^---^
Galanthus nivalis	28	^33.8^	2	^---^	12	^7.9^	.	^---^	.	^---^	4	^---^
Quercus petraea	44	^31.1^	33	^18.6^	19	^1.4^	7	^---^	3	^---^	.	^---^
Campanula trachelium	34	^27.1^	23	^12.5^	21	^9.8^	3	^---^	.	^---^	.	^---^
Anemone nemorosa	18	^26.3^	12	^15.1^	.	^---^	.	^---^	.	^---^	.	^---^
Corylus avellana	24	^26.1^	6	^---^	6	^---^	.	^---^	.	^---^	12	^6.4^
Veratrum nigrum	8	^26^	.	^---^	.	^---^	.	^---^	.	^---^	.	^---^
Serratula tinctoria	12	^22.8^	4	^2.7^	.	^---^	3	^---^	.	^---^	.	^---^
Tilia cordata	36	^15.1^	71	^52.8^	10	^---^	3	^---^	.	^---^	12	^---^
Symphytum tuberosum	34	^9.7^	62	^39.3^	35	^11.2^	9	^---^	3	^---^	4	^---^
Fragaria moschata	.	^---^	12	^32.6^	.	^---^	.	^---^	.	^---^	.	^---^
Primula vulgaris	12	^9.1^	25	^32.1^	4	^---^	.	^---^	.	^---^	.	^---^
Polygonatum multiflorum	4	^---^	17	^27.7^	2	^---^	.	^---^	3	^---^	.	^---^
Salvia glutinosa	12	^7.5^	23	^26^	10	^4.8^	.	^---^	.	^---^	.	^---^
Mercurialis perennis	26	^4.9^	44	^24.2^	40	^19.7^	9	^---^	3	^---^	8	^---^
Melica nutans	2	^---^	17	^22.6^	4	^---^	7	^2.5^	3	^---^	.	^---^
Carex digitata	42	^19.6^	44	^21.4^	21	^---^	16	^---^	10	^---^	8	^---^
Acer monspessulanum	4	^---^	.	^---^	69	^46.6^	21	^---^	23	^---^	28	^4^
Melittis melissophyllum	18	^---^	17	^---^	81	^44.9^	37	^2.9^	26	^---^	24	^---^
Acer campestre	38	^4.1^	48	^13.5^	69	^33.2^	22	^---^	13	^---^	12	^---^
Aristolochia lutea	2	^---^	.	^---^	12	^29.4^	.	^---^	.	^---^	.	^---^
Euonymus europaeus	4	^---^	15	^4.6^	31	^28.1^	8	^---^	10	^---^	.	^---^
Paeonia officinalis	18	^---^	21	^---^	42	^23.4^	16	^---^	23	^2.8^	4	^---^
Vincetoxicum hirundinaria	.	^---^	15	^---^	29	^3.4^	67	^42.2^	36	^10.3^	8	^---^
Lathyrus sylvestris	.	^---^	.	^---^	.	^---^	7	^23.5^	.	^---^	.	^---^
Genista tinctoria	.	^---^	.	^---^	.	^---^	7	^23.5^	.	^---^	.	^---^
Ostrya carpinifolia	.	^---^	6	^---^	4	^---^	22	^20^	15	^9.2^	8	^---^
Lathyrus niger	.	^---^	.	^---^	.	^---^	5	^21^	.	^---^	.	^---^
Dactylorhiza maculata agg.	.	^---^	.	^---^	.	^---^	5	^21^	.	^---^	.	^---^
Campanula persicifolia	.	^---^	.	^---^	.	^---^	5	^21^	.	^---^	.	^---^
Festuca heterophylla	.	^---^	.	^---^	.	^---^	5	^21^	.	^---^	.	^---^
Cotinus coggygria	.	^---^	.	^---^	6	^---^	7	^---^	79	^75.2^	8	^---^
Brachypodium rupestre	.	^---^	4	^---^	46	^15.3^	43	^12.9^	79	^48.1^	8	^---^
Carex humilis	.	^---^	.	^---^	2	^---^	7	^1.7^	26	^38.4^	.	^---^
Prunus spinosa	.	^---^	4	^---^	8	^2.7^	3	^---^	26	^33.5^	.	^---^
Dictamnus albus	.	^---^	4	^---^	8	^---^	14	^3.7^	36	^33.4^	8	^---^
Teucrium chamaedrys	.	^---^	.	^---^	4	^---^	4	^---^	18	^26.5^	4	^---^
Asparagus tenuifolius	2	^---^	6	^---^	12	^5^	9	^---^	26	^25.3^	.	^---^
Silene nutans	.	^---^	.	^---^	.	^---^	3	^1.5^	10	^25^	.	^---^
Galium lucidum	.	^---^	.	^---^	.	^---^	1	^---^	8	^22.8^	.	^---^
Allium sphaerocephalon	.	^---^	.	^---^	.	^---^	.	^---^	5	^20.8^	.	^---^
Melica uniflora	.	^---^	.	^---^	.	^---^	.	^---^	5	^20.8^	.	^---^
Polygonatum odoratum	.	^---^	2	^---^	6	^---^	17	^5.5^	28	^20.3^	24	^14.7^
Moehringia muscosa	12	^---^	2	^---^	17	^---^	.	^---^	5	^---^	76	^65.9^
Asplenium trichomanes	4	^---^	4	^---^	6	^---^	3	^---^	.	^---^	56	^59.9^
Campanula pyramidalis	.	^---^	.	^---^	.	^---^	.	^---^	.	^---^	40	^59.8^
Geranium robertianum	.	^---^	.	^---^	.	^---^	.	^---^	.	^---^	8	^26^
Prunus mahaleb	.	^---^	.	^---^	2	^---^	8	^3^	8	^2.6^	20	^25.3^
Asplenium ruta-muraria	.	^---^	.	^---^	2	^---^	3	^---^	.	^---^	12	^25^
Ajuga reptans	8	^6^	2	^---^	4	^---^	.	^---^	.	^---^	16	^22.4^
Frangula rupestris	.	^---^	2	^---^	10	^---^	16	^5.3^	15	^4.7^	28	^22.1^
Dentaria enneaphyllos	98	^61.5^	77	^41.6^	23	^---^	1	^---^	.	^---^	.	^---^
Lathyrus vernus	48	^39.8^	33	^21.8^	12	^---^	.	^---^	.	^---^	.	^---^
Convallaria majalis	42	^22.8^	56	^38.4^	10	^---^	11	^---^	.	^---^	8	^---^
Hepatica nobilis	54	^31.9^	8	^---^	62	^40.8^	5	^---^	.	^---^	12	^---^
Galeobdolon montanum	46	^26.5^	10	^---^	52	^33.1^	5	^---^	.	^---^	16	^---^
Helleborus multifidus ssp. istriacus	54	^2.5^	88	^32.5^	75	^21.3^	32	^---^	59	^7^	.	^---^
Hedera helix	30	^---^	83	^25.6^	56	^1.2^	53	^---^	23	^---^	84	^26.2^
Sesleria autumnalis	76	^---^	75	^---^	96	^11^	100	^16.7^	100	^16.7^	80	^---^
Fraxinus ornus	70	^---^	88	^13.9^	69	^---^	83	^9.2^	46	^---^	88	^24.4^
Quercus cerris	54	^---^	75	^12.4^	60	^---^	78	^14.8^	46	^---^	56	^---^
Mercurialis ovata	18	^---^	25	^8.5^	27	^10.9^	13	^---^	15	^---^	8	^---^
Quercus pubescens	.	^---^	2	^---^	6	^---^	13	^8.7^	18	^16.6^	8	^---^

**Table 4:** Correlation of flowering onset with topographical and environmental variables. Legend: r—correlation coefficient; p.par—parametric correlation, p.perm.—with permutation test and p.modif—with modified permutation test; calculated probability in interval: ‘***’ 0.001 ‘**’ 0.01 ‘*’ 0.05; p.perm and p.modif. do not apply to topographical variables.

March	r	p.par.	p.perm.	p.modif.	June	r	p.par.	p.perm.	p.modif.
Depth	0.434	***			Depth	−0.361	***		
SVF	−0.107	-			SVF	0.314	***		
Bare rock	−0.046	-			Bare rock	−0.240	***		
Moisture	0.493	***	**	*	Moisture	−0.283	***	**	-
Nutrients	0.636	***	**	**	Nutrients	−0.303	***	**	-
Light	−0.427	***	**	-	Light	0.444	***	**	*
Temperature	−0.214	***	**	-	Temperature	0.298	***	**	-
Reaction	0.099	-	-	-	Reaction	−0.007	-	-	-
**April**	**r**	**p.par.**	**p.perm.**	**p.modif.**	**July**	**r**	**p.par.**	**p.perm.**	**p.modif.**
Depth	0.132	*			Depth	−0.033	-		
SVF	−0.156	**			SVF	0.041	-		
Bare rock	0.059	-			Bare rock	0.001	-		
Moisture	0.060	-	-	-	Moisture	−0.123	*	*	-
Nutrients	0.210	***	**	-	Nutrients	−0.179	**	**	-
Light	−0.229	***	**	-	Light	0.257	***	**	-
Temperature	0.151	*	*	-	Temperature	0.050	-	-	-
Reaction	−0.185	**	**	-	Reaction	0.124	*	*	-
**May**	**r**	**p.par.**	**p.perm.**	**p.modif.**	**August**	**r**	**p.par.**	**p.perm.**	**p.modif.**
Depth	−0.192	*			Depth	−0.025	-		
SVF	0.118	**			SVF	−0.198	***		
Bare rock	−0.018	-			Bare rock	0.237	***		
Moisture	−0.307	***	-	-	Moisture	0.016	-	-	-
Nutrients	−0.331	***	-	-	Nutrients	0.150	*	**	-
Light	0.209	***	-	-	Light	0.007	-	-	-
Temperature	−0.040	-	-	-	Temperature	−0.064	-	-	-
Reaction	0.126	*	*	-	Reaction	−0.052	-	-	-

**Table 5:** List of elaborated dolines. Depth of dolines and length of the transects. Legend: Nr.—indication of doline; Depth (m)—depth of doline in meters; N—length of transect toward the north (in meters); S length of transect toward the south (in meters).

Nr.	Depth (m)	N (North)	S (South)	Nr.	Depth (m)	N (North)	S (South)
1	5.98	14	20	6	13.28	32	36
2	7.51	14	24	7	18.84	34	54
3	8.17	18	34	8	21.74	38	46
4	2.45	10	22	9	13.65	30	30
5	8.71	22	18	10	15.4	40	34

## Data Availability

The original contributions presented in this study are included in this article or the Supplementary Material.

## Supplementary Materials

The following supporting information can be downloaded at https://www.mdpi.com/article/10.3390/plants14071093/s1, Figure S1: Results of silhouette analysis; Table S1: Table of sample plots; Table S2: Details of sample plots and input data for analyses.

## Nomenclature

The nomenclature of plant species follows the Euro+Med Plant Base [Available online: https://www.emplantbase.org, accessed 20 November 2024]. The nomenclature of forest communities follows [80], except *Fraxino orni-Aceretum pseudoplatani* Dakskobler et Poldini 2021, and higher units follow [24].

## Abbreviations

The following abbreviations are used in this manuscript:

DCA	Detrended Correspondence Analysis
EIV	Ellenberg indicator values
SVF	Sky-view factor

## References

[1] Hassan T., Gulzar R., Hamid M., Ahmad R., Waza S.A., Khuroo A.A. (2024). Plant phenology shifts under climate warming: A systematic review of recent scientific literature. Environ. Monit. Assess..

[2] Singh P., Gargi B., Semwal P., Verma S. (2024). Global research and research progress on climate change and their impact on plant phenology: 30 years of investigations through bibliometric analysis. Theor. Appl. Climatol..

[3] Puchałka R., Klisz M., Koniakin S., Czortek P., Dylewski Ł., Paź-Dyderska S., Vítková M., Sádlo J., Rašomavičius V., Čarni A. (2022). Citizen science helps predictions of climate change impact on flowering phenology: A study on *Anemone nemorosa*. Agric. For. Meteorol..

[4] Jiang Q., Yuan Z., Yin J., Yao M., Qin T., Lü X., Wu G. (2024). Response of vegetation phenology to climate factors in the source region of the Yangtze and Yellow Rivers. J. Plant Ecol..

[5] Zhang J., Chen S., Wu Z., Fu Y.H. (2022). Review of vegetation phenology trends in China in a changing climate. Prog. Phys. Geogr. Earth Environ..

[6] Vitasse Y., Baumgarten F., Zohner C.M., Rutishauser T., Pietragalla B., Gehrig R., Dai J., Wang H., Aono Y., Sparks T.H. (2022). The great acceleration of plant phenological shifts. Nat. Clim. Change.

[7] Frei K., Vojtkó A., Farkas T., Erdős L., Barta K., E-Vojtkó A., Tölgyesi C., Bátori Z. (2023). Topographic depressions can provide climate and resource microrefugia for biodiversity. iScience.

[8] Bátori Z., Vojtkó A., Maák I.E., Lőrinczi G., Farkas T., Kántor N., Tanács E., Kiss P.J., Juhász O., Módra G. (2019). Karst dolines provide diverse microhabitats for different functional groups in multiple phyla. Sci. Rep..

[9] Knauf A.E., Litton C.M., Cole R.J., Sparks J.P., Giardina C.P., Gerow K.G., Quiñones-Santiago M. (2021). Nutrient-use strategy and not competition determines native and invasive species response to changes in soil nutrient availability. Restor. Ecol..

[10] Puchałka R., Paź-Dyderska S., Dylewski Ł., Czortek P., Vítková M., Sádlo J., Klisz M., Koniakin S., Čarni A., Rašomavičius V. (2023). Forest herb species with similar European geographic ranges may respond differently to climate change. Sci. Total Environ..

[11] Jakob A., Breg Valjavec M., Čarni A. (2025). Determination of forest communities on the basis of small plots (microplots) within the geomorphologically diverse landscape of the Kras plateau (Italy, Slovenia). For. Ecosyst..

[12] Frei K., E-Vojtkó A., Tölgyesi C., Vojtkó A., Farkas T., Erdős L., Li G., Lőrincz Á., Bátori Z. (2025). Topographic complexity drives trait composition as well as functional and phylogenetic diversity of understory plant communities in microrefugia: New insights for conservation. For. Ecosyst..

[13] Trew B.T., Maclean I.M.D. (2021). Vulnerability of global biodiversity hotspots to climate change. Glob. Ecol. Biogeogr..

[14] Hong P., Schmid B., De Laender F., Eisenhauer N., Zhang X., Chen H., Craven D., De Boeck H.J., Hautier Y., Petchey O.L. (2022). Biodiversity promotes ecosystem functioning despite environmental change. Ecol. Lett..

[15] Zhuo Y., Wang M., Koirala S., Hughes A.C., Xu W., Abdulnazar A., Rajabi A.M., Davletbakov A., Haider J., Khan M.Z. (2025). Considering Plant-Ungulate Interaction Contribute to Maximizing Conservation Efficiency under Climate Change. Glob. Ecol. Conserv..

[16] Wakita K., Obara H., Oyama N., Murakami T. (2025). Reassessing the Global Significance of Geological Heritage in the Miné-Akiyoshidai Karst Plateau Aspiring UNESCO Global Geopark. Geosciences.

[17] Creati N., Paganini P., Sterzai P., Pavan A. (2025). Mapping of karst sinkholes from LIDAR data using machine-learning methods in the Trieste area. J. Spat. Sci..

[18] Bátori Z., Erdős L., Gajdács M., Barta K., Tobak Z., Frei K., Tölgyesi C. (2021). Managing climate change microrefugia for vascular plants in forested karst landscapes. For. Ecol. Manag..

[19] Pakgohar N., Lengyel A., Botta-Dukát Z. (2024). Quantitative evaluation of internal cluster validation indices using binary data sets. J. Veg. Sci..

[20] Lengyel A., Roberts D.W., Botta-Dukát Z. (2021). Comparison of silhouette-based reallocation methods for vegetation classification. J. Veg. Sci..

[21] Čarni A., Čonč Š., Valjavec M.B. (2022). Landform-vegetation units in karstic depressions (dolines) evaluated by indicator plant species and Ellenberg indicator values. Ecol. Indic..

[22] Tichý L., Chytrý M., Botta-Dukát Z. (2014). Semi-supervised classification of vegetation: Preserving the good old units and searching for new ones. J. Veg. Sci..

[23] Chytrý M., Tichý L., Hennekens S.M., Knollová I., Janssen J.A.M., Rodwell J.S., Peterka T., Marcenò C., Landucci F., Danihelka J. (2020). EUNIS habitat classification: Expert system, characteristic species combinations and distribution maps of European habitats. Appl. Veg. Sci..

[24] Mucina L., Bültmann H., Dierßen K., Theurillat J., Raus T., Čarni A., Šumberová K., Willner W., Dengler J., García R.G. (2016). Vegetation of Europe: Hierarchical floristic classification system of vascular plant, bryophyte, lichen, and algal communities. Appl. Veg. Sci..

[25] Bátori Z., Vojtkó A., Farkas T., Szabó A., Havadtői K., Vojtkó A.E., Tölgyesi C., Cseh V., Erdős L., Maák I.E. (2017). Large- and small-scale environmental factors drive distributions of cool-adapted plants in karstic microrefugia. Ann. Bot..

[26] Hinze J., Albrecht A., Michiels H.-G. (2023). Climate-Adapted Potential Vegetation—A European Multiclass Model Estimating the Future Potential of Natural Vegetation. Forests.

[27] Poldini L. (1989). La Vegetazione del Carso Isontino e Triestino.

[28] Čarni A., Marinček L., Seliškar A., Zupančič M. (2002). Vegetation Map of Forest Communities of Slovenia in Scale 1:400.000.

[29] Klotz S., Kühn I., Durka W. (2002). BIOLFLOR—Eine Datenbank zu Biologisch-Ökologischen Merkmalen der Gefäßpflanzen in Deutschland.

[30] Kattge J., Bönisch G., Díaz S., Lavorel S., Prentice I.C., Leadley P., Tautenhahn S., Werner G.D.A., Aakala T., Abedi M. (2020). TRY plant trait database—Enhanced coverage and open access. Glob. Change Biol..

[31] Martinčič A. (2007). Mala Flora Slovenije.

[32] Lee Z., Kim S., Choi S.J., Joung E., Kwon M., Park H.J., Shim J.S. (2023). Regulation of flowering time by environmental factors in plants. Plants.

[33] Stemkovski M., Bell J.R., Ellwood E.R., Inouye B.D., Kobori H., Lee S.D., Lloyd-Evans T., Primack R.B., Templ B., Pearse W.D. (2023). Disorder or a new order: How climate change affects phenological variability. Ecology.

[34] Breg Valjavec M., Čarni A., Žlindra D., Zorn M., Marinšek A. (2022). Soil organic carbon stock capacity in karst dolines under different land uses. CATENA.

[35] Nojarov P., Stefanov P., Stefanova D., Jelev G. (2024). Climate change, anthropogenic pressure, and sustainable development of karst geosystems (a case study of the Brestnitsa karst geosystem in northern Bulgaria). Sustainability.

[36] Cubino J.P., Biurrun I., Bonari G., Braslavskaya T., Font X., Jandt U., Jansen F., Rašomavičius V., Škvorc Ž., Willner W. (2021). The leaf economic and plant size spectra of European forest understory vegetation. Ecography.

[37] Košir P., Čarni A., Di Pietro R. (2008). Classification and phytogeographical differentiation of broad-leaved ravine forests in southeastern Europe. J. Veg. Sci..

[38] Chapman L., Thornes J.E. (2004). Real-Time Sky-View Factor Calculation and Approximation. J. Atmos. Ocean. Technol..

[39] Stefanovski S., Kokalj Ž., Stepišnik U. (2024). Sky-view factor enhanced doline delineation: A comparative methodological review based on case studies in Slovenia. Geomorphology.

[40] Marcin M., Raschmanová N., Miklisová D., Šupinský J., Kaňuk J., Kováč L. (2024). Karst landforms as microrefugia for soil Collembola: Open versus forested dolines. Elem. Sci. Anthr..

[41] Jakob A., Breg Valjavec M., Čarni A. (2022). Turnover of plant species on an ecological gradient in karst dolines is reflected in plant traits: Chorotypes, life forms, plant architecture and strategies. Diversity.

[42] Marcenò C., Gristina A.S., Chytrý M., Garfi G., Ilardi V., Jiménez-Alfaro B., Paliaga S., Pasta S., Venanzoni R., Guarino R. (2024). Two new grassland associations from the Madonie Mountains (Sicily) disclose critical classification issues in endemic-rich oromediterranean plant communities of the classes *Molinio-Arrhenatheretea* and *Rumici-Astragaletea siculi*. Tuexenia.

[43] Lorer E., Verheyen K., Blondeel H., De Pauw K., Sanczuk P., De Frenne P., Landuyt D. (2024). Forest understorey flowering phenology responses to experimental warming and illumination. New Phytol..

[44] Timerman D., Barrett S.C.H. (2021). The biomechanics of pollen release: New perspectives on the evolution of wind pollination in angiosperms. Biol. Rev..

[45] Prieto P., Penuelas J., Ogaya R., Estiarte M. (2008). Precipitation-dependent flowering of *Globularia alypum* and *Erica multiflora* in Mediterranean shrubland under experimental drought and warming, and its Inter-annual variability. Ann. Bot..

[46] Bogdanović S., Anačkov G., Ćato S., Borovečki-Voska L., Salmeri C., Brullo S. (2024). Allium dinaricum (Amaryllidaceae), a new species of *A*. sect*. Codonoprasum* from the Balkan Peninsula based on morphology and karyology. Willdenowia.

[47] Surina B., Balant M., Glasnović P., Radosavljević I., Fišer Ž., Fujs N., Castro S. (2023). Population size as a major determinant of mating system and population genetic differentiation in a narrow endemic chasmophyte. BMC Plant Biol..

[48] De Frenne P. (2023). Novel light regimes in European forests. Nat. Ecol. Evol..

[49] Ferreiro-Lera G.-B., Penas Á., del Río S. (2024). Unveiling deviations from IPCC temperature projections through Bayesian downscaling and assessment of CMIP6 general circulation models in a climate-vulnerable region. Remote Sens..

[50] Lorente C., Corell D., Estrela M.J., Miró J.J., Orgambides-García D. (2024). Evolution of bioclimatic belts in Spain and the Balearic islands (1953–2022). Climate.

[51] Keppel G., Stralberg D., Morelli T.L., Bátori Z. (2024). Managing climate-change refugia to prevent extinctions. Trends Ecol. Evol..

[52] Rudolf A., Vreš B., Dakskobler I. (2019). Sites of rare form of auricula (*Primula auricula* var. tolminensis nom. prov.) in the southern Julian Alps. Folia Biol. Geol..

[53] Mihevc A., Prelovšek M., Zupan Hajna N. (2010). Introduction to the Dinaric Karst. J. Chem. Inf. Model..

[54] Čarni A. (2019). Overview of Forest Communities in Slovenia.

[55] Vrščaj B., Repe B., Simončič P. (2017). The Soils of Slovenia.

[56] Cervellini M., Zannini P., Di Musciano M., Fattorini S., Jiménez-Alfaro B., Rocchini D., Field R., Vetaas O.R., Irl S.D., Beierkuhnlein C. (2020). A grid-based map for the biogeographical regions of Europe. Biodivers. Data J..

[57] Ford D.C., Williams P. (2007). Karst Hydrogeology and Geomorphology.

[58] Cernatič-Gregorič A., Zega M. (2010). The impact of human activities on dolines (sinkholes)—Typical geomorphologic features on Karst (Slovenia) and possibilities of their preservation. Geogr. Panon..

[59] Kranjc A. (2013). Classification of Closed Depressions in Carbonate Karst. Treatise Geomorphol..

[60] Aguilon D.J., Vojtkó A., Tölgyesi C., Erdős L., Kiss P.J., Lőrinczi G., Juhász O., Frei K., Bátori Z. (2020). Karst environments and disturbance: Evaluation of the effects of human activity on grassland and forest naturalness in dolines. Biologia.

[61] Kiss P.J., Tölgyesi C., Bóni I., Erdős L., Vojtkó A., Maák I.E., Bátori Z. (2020). The effects on intensive logging on the capacity of karst dolines to provide potential microrefugia for cool-adapted plants. Acta Geogr. Slov..

[62] Braun-Blanquet J. (1964). Pflanzensoziologie, Grundzüge der Vegetationskunde.

[63] Čonč Š., Oliveira T., Belotti E., Bufka L., Černe R., Heurich M., Valjavec M.B., Krofel M. (2024). Revealing functional responses in habitat selection of rocky features and rugged terrain by Eurasian lynx (*Lynx lynx*) using LiDAR data. Landsc. Ecol..

[64] Oksanen J., Simpson G., Blanchet F., Kindt R., Legendre P., Michin P., O’Hara R.B., Solymos P., Stevens H.M.H., Szöcs E. Vegan: Community Ecology Package, R Package version 2.6-4. https://github.com/vegandevs/vegan.

[65] Maechler M., Rousseeuw P., Hubert M., Hornik K. (2023). cluster: Cluster Analysis Basics and Extensions. R Package Version 2.1.4. https://CRAN.R-project.org/package=cluster.

[66] R Core Team R: A Language and Environment for Statistical Computing. R Foundation for Statistical Computing, version 3.6.1. https://www.R-project.org..

[67] Tichý L. (2002). JUICE, software for vegetation classification. J. Veg. Sci..

[68] Environmental Systems Research Institute (2024). ArcInfo (Version 10.8) [Computer Software]. https://www.esri.com.

[69] Wickham H. (2016). Ggplot2: Elegant Graphics for Data Analysis.

[70] Tichý L., Axmanová I., Dengler J., Guarino R., Jansen F., Midolo G., Nobis M.P., Van Meerbeek K., Aćić S., Attorre F. (2023). Ellenberg-type indicator values for European vascular plant species. J. Veg. Sci..

[71] Tölgyesi C., Bátori Z., Erdos L. (2014). Using statistical tests on relative ecological indicator values to compare vegetation units—Different approaches and weighting methods. Ecol. Indic..

[72] Pignatti S., Menegoni P., Pietrosanti S. (2005). Valori di bioindicazione delle piante vascolari. Valori di indicatione secondo Ellenberg per la piante delle flora d’Italia. Braun-Blanquetia.

[73] Chytrý M., Tichý L., Holt J., Botta-Dukát Z. (2002). Determination of diagnostic species with statistical fidelity measures. J. Veg. Sci..

[74] Tichý L., Chytrý M. (2006). Statistical determination of diagnostic species for site groups of unequal size. J. Veg. Sci..

[75] Hill M.O., Gauch H.G. (1980). Detrended correspondence analysis: An improved ordination technique. Vegetatio.

[76] Zelený D. (2018). Which results of the standard test for community-weighted mean approach are too optimistic?. J. Veg. Sci..

[77] Zelený D., Schaffers A.P. (2012). Too good to be true: Pitfalls of using mean Ellenberg indicator values in vegetation analyses. J. Veg. Sci..

[78] ter Braak C.J.F., Šmilauer P. (2012). CANOCO Reference Manual and User’s Guide: Software for Ordination (Version 5.0).

[79] Guralnick R., Crimmins T., Grady E., Campbell L. (2024). Phenological response to climatic change depends on spring warming velocity. Commun. Earth Environ..

[80] Šilc U., Čarni A. (2012). Conspectus of vegetation syntaxa in Slovenia. Hacquetia.

