# Supplementary material for: Phenological Plant Pattern in the Topographic Complex Karstic Landscape of the Northern Dinaric Alps
Source: Plants (Basel). 2025 Apr 1;14(7):1093. doi: 10.3390/plants14071093 (PMC11991487; doi:10.3390/plants14071093)
Supplement: Supplementary file 1 [file plants-14-01093-s001.zip › Supplemenatry material Figure S1.pdf]

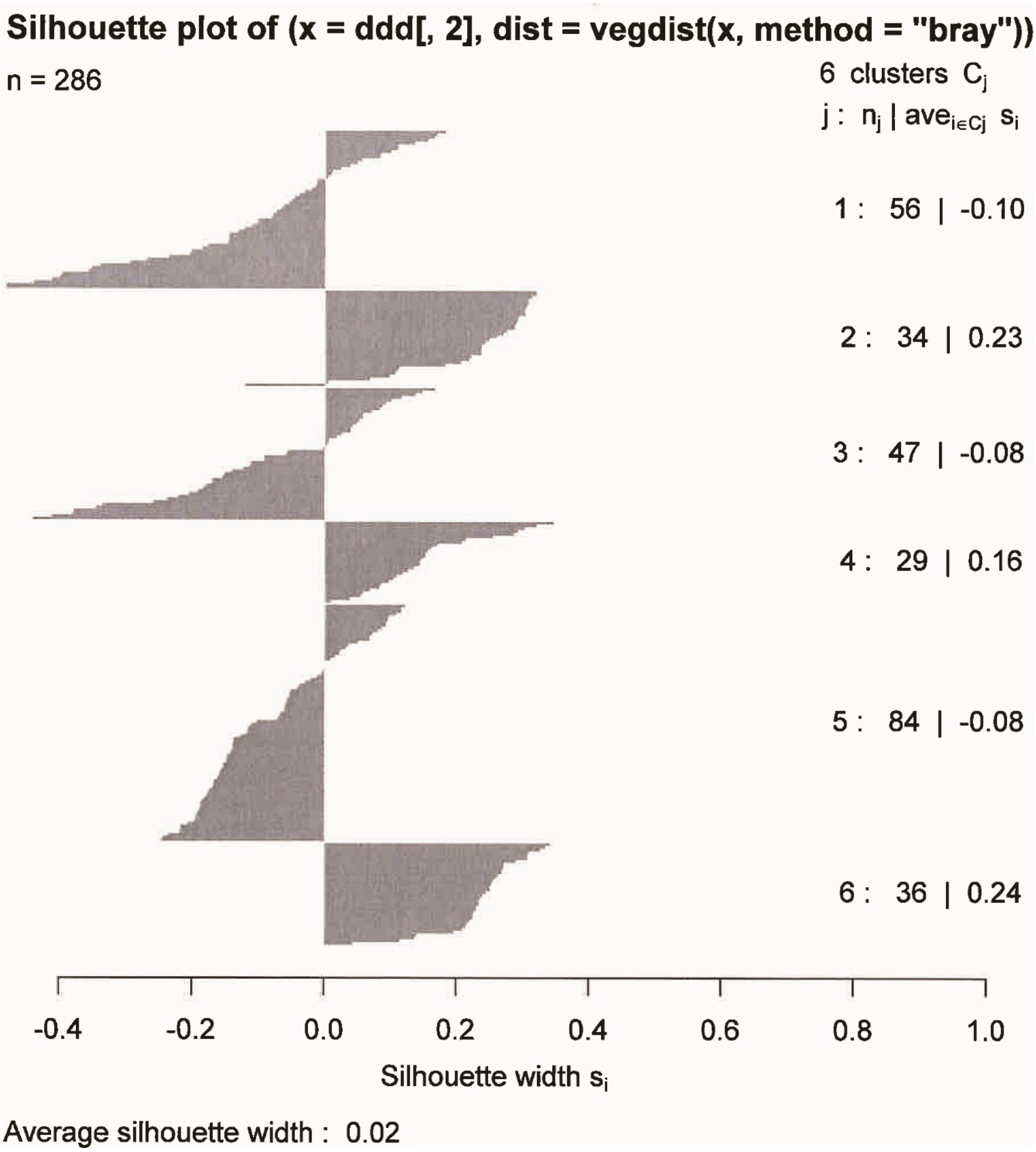

Figure S1. Silhouette analysis suggested that the optimal division of the matrix would be into six groups according to k-means clustering.
